# [Cu_2_(HF_2_)(H_2_O)_8_][FeF_6_]·2H_2_O

**DOI:** 10.1107/S1600536809006874

**Published:** 2009-03-06

**Authors:** Armel Le Bail, Anne-Marie Mercier

**Affiliations:** a Laboratoire des Oxydes et Fluorures, CNRS UMR 6010, Université du Maine, 72085 Le Mans, France

## Abstract

The title compound, octaaqua­(hydrogenfluorido)dicopper(II) hexa­fluoridoferrate(III) dihydrate, was synthesized under hydro­thermal conditions. The Cu atom is coordinated by one F and five O atoms within a highly distorted octa­hedron, forming dimeric [Cu_2_(H_2_O)_8_HF_2_]^3+^ units by edge sharing. These units are hydrogen bonded to [FeF_6_]^3−^ anions and to an inter­stitial water mol­ecule. The former feature Fe^3+^ on a special position (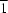). The dimeric copper units are linked to adjacent dimers by F—H⋯F hydrogen bonds. Additional O—H⋯O and O—H⋯F hydrogen bonds help to consolidate the crystal packing.

## Related literature

For other hydrated copper-iron fluorides, see: Kummer & Babel (1987 ▶); Leblanc & Ferey (1990 ▶). For F⋯F distances, see: Frevel & Rinn (1962 ▶); Massa & Herdtweck (1983 ▶). For asymmetrical F—H⋯F hydrogen bonding, see: Roesky *et al.* (1990 ▶); Gerasimenko *et al.* (2007 ▶); Gerken *et al.* (2002 ▶). For Pb_8_MnFe_2_F_24_, see: Le Bail & Mercier (1992 ▶). For valence-bond analysis, see: Brown & Altermatt (1985 ▶); Brese & O’Keeffe (1991 ▶).

## Experimental

### 

#### Crystal data

[Cu_2_(HF_2_)(H_2_O)_8_][FeF_6_]·2H_2_O
*M* *_r_* = 516.12
Triclinic, 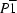

*a* = 6.659 (2) Å
*b* = 7.450 (3) Å
*c* = 8.377 (5) Å
α = 107.37 (4)°
β = 106.89 (5)°
γ = 94.26 (3)°
*V* = 373.6 (3) Å^3^
*Z* = 1
Mo *K*α radiation
μ = 3.91 mm^−1^
*T* = 293 K
0.11 × 0.10 × 0.05 mm

#### Data collection

Siemens AED2 diffractometer
Absorption correction: gaussian (*SHELX76*; Sheldrick, 2008 ▶) *T*_min_ = 0.698, *T*_max_ = 0.845
3303 measured reflections
3303 independent reflections
2044 reflections with *I* > 2σ(*I*)
3 standard reflections frequency: 120 min intensity decay: 15%

#### Refinement

*R*[*F* ^2^ > 2σ(*F* ^2^)] = 0.040
*wR*(*F* ^2^) = 0.080
*S* = 1.00
3303 reflections
133 parameters
15 restraints
H atoms treated by a mixture of independent and constrained refinement
Δρ_max_ = 0.62 e Å^−3^
Δρ_min_ = −0.63 e Å^−3^

### 

Data collection: *STADI4* (Stoe & Cie, 1998 ▶); cell refinement: *STADI4*; data reduction: *X-RED* (Stoe & Cie, 1998 ▶); program(s) used to solve structure: *SHELXS97* (Sheldrick, 2008 ▶); program(s) used to refine structure: *SHELXL97* (Sheldrick, 2008 ▶); molecular graphics: *DIAMOND* (Brandenburg, 1999 ▶) and *ORTEP-3* (Farrugia, 1997 ▶); software used to prepare material for publication: *publCIF* (Westrip, 2009 ▶).

## Supplementary Material

Crystal structure: contains datablocks I, global. DOI: 10.1107/S1600536809006874/fi2069sup1.cif

Structure factors: contains datablocks I. DOI: 10.1107/S1600536809006874/fi2069Isup2.hkl

Additional supplementary materials:  crystallographic information; 3D view; checkCIF report

## Figures and Tables

**Table 1:** Selected bond lengths (Å)

Cu—F4	1.9058 (19)
Cu—O1	1.939 (2)
Cu—O2	1.958 (2)
Cu—O3	1.977 (2)
Cu—O4	2.349 (3)
Fe—F1	1.9204 (16)
Fe—F2^i^	1.930 (2)
Fe—F3	1.9402 (17)
Cu—O3^ii^	2.715 (3)
Cu⋯Cu^ii^	3.575 (3)

Symmetry codes: (i) 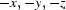; (ii) 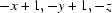.

**Table 2:** Hydrogen-bond geometry (Å, °)

*D*—H⋯*A*	*D*—H	H⋯*A*	*D*⋯*A*	*D*—H⋯*A*
O1—H11⋯F1^iii^	1.00 (4)	1.59 (4)	2.590 (3)	173 (4)
O1—H12⋯F2	0.99 (4)	1.71 (4)	2.679 (3)	167 (4)
O2—H21⋯F1^ii^	1.02 (5)	1.61 (5)	2.595 (3)	161 (4)
O2—H22⋯O5^iv^	0.98 (4)	1.82 (4)	2.699 (3)	147 (5)
O3—H31⋯F3^ii^	1.07 (5)	1.52 (5)	2.577 (2)	167 (5)
O3—H32⋯F3^v^	1.04 (5)	1.64 (5)	2.668 (3)	171 (5)
O4—H41⋯F2^vi^	1.02 (4)	2.07 (9)	2.766 (3)	123 (8)
O4—H42⋯O5^v^	1.01 (9)	1.81 (9)	2.793 (3)	164 (8)
O5—H51⋯F2^vii^	1.01 (6)	2.05 (7)	2.888 (3)	139 (5)
O5—H51⋯F3^viii^	1.01 (6)	2.23 (5)	3.072 (4)	140 (6)
O5—H52⋯F4	1.05 (7)	1.63 (7)	2.676 (3)	175 (6)
F4—H6⋯F4^vi^	0.91	1.82	2.597 (4)	142

Symmetry codes: (ii) 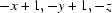; (iii) 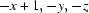; (iv) 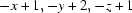; (v) 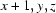; (vi) 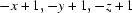; (vii) 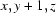; (viii) 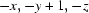.

**Table 3:** Valence-bond analysis according to the empirical expression from Brown & Altermatt (1985 ▶), using parameters for solids from Brese & O’Keeffe (1991 ▶)

	O1	O2	O3	O4	F4	F1	F2	F3	O5	Σ	Σ_expected_
Cu	0.49	0.47	0.45	0.16	0.44					2.01	2
Fe						0.51 (× 2)	0.49 (× 2)	0.48 (× 2)		2.96	3
H11	0.80					0.20				1	1
H12	0.80						0.20			1	1
H21		0.80				0.20				1	1
H22		0.80							0.20	1	1
H31			0.80					0.20		1	1
H41				0.80			0.20			1	1
H42				0.80					0.20	1	1
H51							0.10	0.10	0.80	1	1
H52					0.20				0.80	1	1
H6					0.80 (× 0.5)					1	1
					0.20 (× 0.5)						
Σ	2.09	2.07	2.05	1.76	1.14	0.91	0.99	0.98	2.00		
Σ_expected_	2	2	2	2	1	1	1	1	2		

**Fig. 1.**
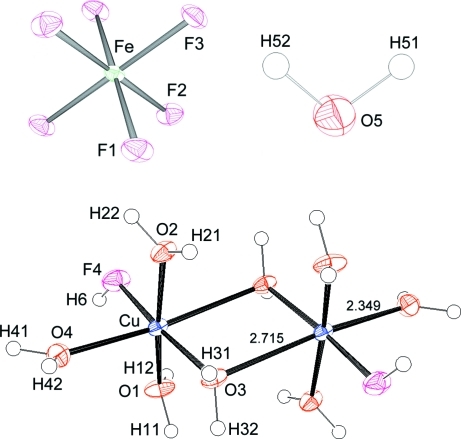
ORTEP-3 view (Farrugia, 1997) of the FeF63- octahedron, the isolated water molecule and the Cu(H2O)4F+ square-based pyramid which is forming octahedra dimeric units when considering the very long Cu-O3 distance (2.715Å). Displacement ellipsoids are shown at the 50% probability level. Symmetry-equivalent F atoms in the FeF6 group are generated by (i) -x, -y, -z. Symmetry-equivalent atoms in the [Cu2(H2O)8HF2]3+ unit are generated by (iii) 1-x, 1-y, -z.

**Fig. 2.**
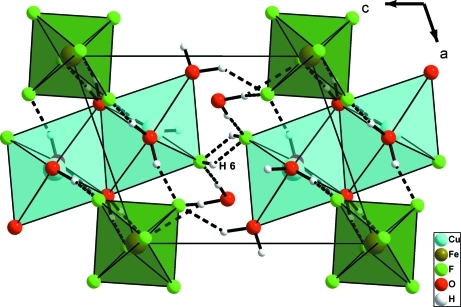
Crystal packing with view along [010]. Hydrogen bonding is shown as dashed lines. Copper coordination shown as distorted octahedra (adding O(3) at 2.715 Å from Cu) sharing an edge.

**Fig. 3.**
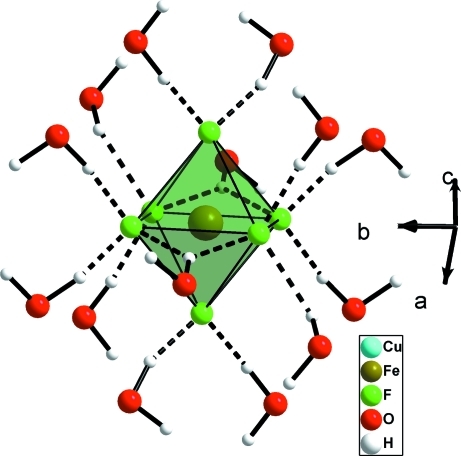
The 14 water molecules, from the Cu coordination sphere or isolated, involved in hydrogen bonding with the FeF63- octahedron.

## supplementary crystallographic information

### Comment

[Cu(H_2_O)_4_H_0.5_F]_2_(FeF_6_)(H_2_O)_2_ is the third hydrated copper–iron fluoride, the two previous ones being Cu_3_Fe_2_F_12_(H_2_O)_12_ (Kummer & Babel, 1987) and CuFe_2_F_8_(H_2_O)_2_ (Leblanc & Ferey, 1990). In this new compound, an almost perfect (FeF_6_)^3-^ octahedron, placed on an inversion center, is connected exclusively by hydrogen bonding to edge-sharing bi-octahedral [Cu_2_(H_2_O)_8_HF_2_]^3+^ units and to an interstitial water molecule. The copper atom can be considered to be five-coordinated by four water molecules and one F atom in the fashion of a square pyramid (Figure 1). The square is constituted by the F atom and three water molecules at distances in the 1.906–1.977 Å range (Table 1), whereas Ow(4), at the top of the pyramid is at 2.349 Å (Jahn-Teller distortion). However, if one considers the next neighbour Ow(3) at 2.715 Å as sixth ligand, thecoordination can be described as very distorted octahedral, yielding centrosymmetric, dimeric units with a Cu—Cu distance of 3.575 Å. The dimers are bonded to adjacent dimers through F(4) by a HF_2_^-^ ion (figure 2), however the F—F distance (2.597 Å) is much larger (Table 2) than usually observed (2.27 Å in LiHF_2_ (Frevel & Rinn, 1962), 2.28 in BaHF_3_ (Massa & Herdtweck, 1983). Moreover, the hydrogen atom H(6) which would have been expected at the 1/2, 1/2, 1/2 special position, exactly at the middle of the F(4)—F(4) atoms, in order to form a linear (F—H—F)^-^ ion, was seen on the Fourier difference map as occupying more probably half a general position. It is then more a F—H···F bridge than a F—H—F one. Such asymmetrical F—H···F hydrogen bonding was observed in many cases for F—F distances going up to 2.686 Å in [(*η*^5^-C_5_Me_5_)NbF_4_(HF)AsF_3_]_2_ (Roesky *et al.*, 1990), 2.429 to 2.512 Å in [OsO_3_F](HF)_2_[AsF_6_] (Gerken *et al.*, 2002), 2.326 to 2.402 Å in Rb_2-_*_x_*K*_x_*ZrF_6_(HF)_2_ (Gerasimenko *et al.*, 2007).

The charge of the cations is balanced by the centrosymmetric anion [FeF_6_]^3–^. There are 14 water molecules around the (FeF_6_)^3-^ octahedron, the H(51) atom corresponds to a bifurcated hydrogen bond towards F(2) and F(3) (figure 3). This helps the bond valence calculations to provide relatively satisfying results (Table 3), the largest disagreements being on O(4) and F(4). The contribution from the long Cu—Ow(3) distance is negligible.

Comparing with the Cu_3_Fe_2_F_12_(H_2_O)_12_ crystal structure (Kummer & Babel, 1987), also triclinic, there are strong differences. The three copper atoms and two Fe atoms are all placed on inversion centers. One (FeF_6_)^3-^ octahedron is isolated, and the other forms chiolite-like square meshes by sharing F corners with tetra-hydrated Cu(H_2_O)_4_F_2_ elongated octahedra (the long distances are the two Cu—F ones, close to 2.32 Å for the three independent copper sites). In CuFe_2_F_8_(H_2_O)_2_ (Leblanc & Ferey, 1990), the CuF_4_(H_2_O)_2_ octahedra show two long Cu—F distances (2.451 Å). So, in both cases, the long distances are Cu—F ones, which is not the case of the title compound.

A study of the magnetic properties is currently in progress.

### Experimental

Hydrothermal growth at 493 K from (2PbF_2_/2CuF_2_/FeF_3_) in HF 5*M* or 1*M* solutions, produced large crystals which could be identified as corresponding to Cu_3_Fe_2_F_12_(H_2_O)_12_ (Kummer & Babel, 1987) (5*M* solution) or to the title compound (1*M*). Both compounds are occurring with the same intense blue color and could have been confused if no powder pattern had been recorded. At other starting compositions, mixtures of both compounds could be observed, and also together especially with Pb_8_CuFe_2_F_24_ (starting from 2PbF_2_/CuF_2_/2FeF_3_ for instance), isostructural with Pb_8_MnFe_2_F_24_ (Le Bail & Mercier, 1992).

### Refinement

The hydrogen atoms were all located on the difference Fourier map. Those of the water molecules were restrained to have Ow—H and H—H distances respectively close to 1.0 and 1.59 Å, their *U*_iso_ values were refined by groups of two. The hydrogen atom of the HF group was let at its difference Fourier map position with a fixed *U*_iso_.

### Crystal data

**Table:**

[Cu_2_(H_2_F)(H_2_O)_8_][FeF_6_]·2H_2_O	*Z* = 1
*M**_r_* = 516.12	*F*(000) = 257
Triclinic, *P*1	*D*_x_ = 2.294 Mg m^−^^3^
Hall symbol: -P 1	Mo *K*α radiation, λ = 0.71069 Å
*a* = 6.659 (2) Å	Cell parameters from 28 reflections
*b* = 7.450 (3) Å	θ = 2.7–35°
*c* = 8.377 (5) Å	µ = 3.91 mm^−^^1^
α = 107.37 (4)°	*T* = 293 K
β = 106.89 (5)°	Platelet, blue
γ = 94.26 (3)°	0.11 × 0.10 × 0.05 mm
*V* = 373.6 (3) Å^3^	

### Data collection

**Table:**

Siemens AED2 diffractometer	2044 reflections with *I* > 2σ(*I*)
Radiation source: fine-focus sealed tube	*R*_int_ = 0.0000
graphite	θ_max_ = 35.0°, θ_min_ = 2.7°
2θ/ω scans	*h* = −10→10
Absorption correction: gaussian (*SHELX76*; Sheldrick, 2008)	*k* = −12→11
*T*_min_ = 0.698, *T*_max_ = 0.845	*l* = 0→13
3303 measured reflections	3 standard reflections every 120 min
3303 independent reflections	intensity decay: 15%

### Refinement

**Table:**

Refinement on *F*^2^	Secondary atom site location: difference Fourier map
Least-squares matrix: full	Hydrogen site location: difference Fourier map
*R*[*F*^2^ > 2σ(*F*^2^)] = 0.040	H atoms treated by a mixture of independent and constrained refinement
*wR*(*F*^2^) = 0.080	*w* = 1/[σ^2^(*F*_o_^2^) + (0.03*P*)^2^] where *P* = (*F*_o_^2^ + 2*F*_c_^2^)/3
*S* = 1.00	(Δ/σ)_max_ = 0.004
3303 reflections	Δρ_max_ = 0.62 e Å^−^^3^
133 parameters	Δρ_min_ = −0.63 e Å^−^^3^
15 restraints	Extinction correction: *SHELXL97* (Sheldrick, 2008), Fc^*^=kFc[1+0.001xFc^2^λ^3^/sin(2θ)]^-1/4^
Primary atom site location: structure-invariant direct methods	Extinction coefficient: 0.0072 (17)

### Special details

**Table:**

Geometry. All e.s.d.'s (except the e.s.d. in the dihedral angle between two l.s. planes) are estimated using the full covariance matrix. The cell e.s.d.'s are taken into account individually in the estimation of e.s.d.'s in distances, angles and torsion angles; correlations between e.s.d.'s in cell parameters are only used when they are defined by crystal symmetry. An approximate (isotropic) treatment of cell e.s.d.'s is used for estimating e.s.d.'s involving l.s. planes.
Refinement. Refinement of *F*^2^ against ALL reflections. The weighted *R*-factor *wR* and goodness of fit *S* are based on *F*^2^, conventional *R*-factors *R* are based on *F*, with *F* set to zero for negative *F*^2^. The threshold expression of *F*^2^ > σ(*F*^2^) is used only for calculating *R*-factors(gt) *etc*. and is not relevant to the choice of reflections for refinement. *R*-factors based on *F*^2^ are statistically about twice as large as those based on *F*, and *R*- factors based on ALL data will be even larger.

### Fractional atomic coordinates and isotropic or equivalent isotropic displacement parameters (Å^2^)

**Table:**

	*x*	*y*	*z*	*U*_iso_*/*U*_eq_	Occ. (<1)
Cu	0.60473 (5)	0.55727 (4)	0.23479 (4)	0.01662 (8)	
Fe	0.0000	0.0000	0.0000	0.01433 (10)	
F1	0.2310 (3)	−0.0246 (2)	−0.0944 (2)	0.0270 (3)	
F2	0.2029 (3)	0.1318 (2)	0.2331 (2)	0.0275 (3)	
F3	−0.0278 (3)	0.2400 (2)	−0.0437 (2)	0.0272 (4)	
F4	0.4365 (3)	0.5885 (3)	0.3872 (2)	0.0338 (4)	
O1	0.5648 (3)	0.2890 (3)	0.2113 (3)	0.0283 (4)	
O2	0.5992 (3)	0.8217 (3)	0.2411 (3)	0.0244 (4)	
O3	0.7470 (3)	0.5094 (3)	0.0530 (3)	0.0219 (4)	
O4	0.9139 (3)	0.6601 (3)	0.4849 (3)	0.0262 (4)	
O5	0.2376 (4)	0.8884 (3)	0.4476 (3)	0.0291 (4)	
H11	0.641 (6)	0.188 (5)	0.158 (6)	0.064 (10)*	
H12	0.424 (5)	0.226 (6)	0.201 (6)	0.064 (10)*	
H21	0.667 (7)	0.876 (7)	0.167 (5)	0.084 (12)*	
H22	0.604 (8)	0.935 (5)	0.341 (5)	0.084 (12)*	
H31	0.866 (7)	0.623 (6)	0.067 (8)	0.117 (16)*	
H32	0.829 (8)	0.398 (6)	0.022 (8)	0.117 (16)*	
H41	0.937 (15)	0.679 (15)	0.615 (5)	0.24 (3)*	
H42	1.049 (10)	0.734 (13)	0.487 (12)	0.24 (3)*	
H51	0.211 (11)	0.909 (10)	0.330 (6)	0.16 (2)*	
H52	0.315 (12)	0.770 (8)	0.430 (10)	0.16 (2)*	
H6	0.4219	0.4979	0.4375	0.080*	0.50

### Atomic displacement parameters (Å^2^)

**Table:**

	*U*^11^	*U*^22^	*U*^33^	*U*^12^	*U*^13^	*U*^23^
Cu	0.02080 (15)	0.01342 (14)	0.01901 (15)	0.00339 (11)	0.01121 (12)	0.00561 (11)
Fe	0.0157 (2)	0.0129 (2)	0.0174 (2)	0.00267 (17)	0.00901 (19)	0.00591 (18)
F1	0.0276 (8)	0.0268 (8)	0.0391 (9)	0.0100 (6)	0.0248 (7)	0.0139 (7)
F2	0.0255 (8)	0.0296 (8)	0.0217 (8)	−0.0025 (6)	0.0064 (6)	0.0038 (6)
F3	0.0295 (8)	0.0181 (7)	0.0442 (10)	0.0077 (6)	0.0185 (8)	0.0176 (7)
F4	0.0475 (11)	0.0338 (9)	0.0375 (10)	0.0184 (8)	0.0290 (9)	0.0192 (8)
O1	0.0285 (10)	0.0151 (8)	0.0470 (13)	0.0038 (7)	0.0227 (9)	0.0086 (8)
O2	0.0386 (11)	0.0138 (8)	0.0261 (10)	0.0040 (7)	0.0177 (8)	0.0074 (7)
O3	0.0270 (9)	0.0179 (8)	0.0281 (10)	0.0056 (7)	0.0191 (8)	0.0079 (7)
O4	0.0243 (9)	0.0267 (10)	0.0261 (10)	0.0042 (8)	0.0076 (8)	0.0074 (8)
O5	0.0359 (11)	0.0279 (10)	0.0264 (10)	0.0109 (9)	0.0128 (9)	0.0093 (8)

### Geometric parameters (Å, °)

**Table:**

Cu—F4	1.9058 (19)	F2—O5^v^	2.888 (3)
Cu—O1	1.939 (2)	F3—O3^iii^	2.577 (2)
Cu—O2	1.958 (2)	F3—O3^vi^	2.668 (3)
Cu—O3	1.977 (2)	F3—O5^vii^	3.072 (4)
Cu—O4	2.349 (3)	F4—F4^iv^	2.597 (4)
Fe—F1	1.9204 (16)	F4—O1	2.632 (3)
Fe—F2^i^	1.930 (2)	F4—O5	2.676 (3)
Fe—F3	1.9402 (17)	F4—O2	2.730 (3)
F1—O1^ii^	2.590 (3)	F4—O4	3.006 (3)
F1—O2^iii^	2.595 (3)	O1—O3	2.804 (3)
F1—F2	2.709 (3)	O2—O5^viii^	2.699 (3)
F1—F3^i^	2.728 (2)	O2—O3	2.814 (3)
F1—F3	2.732 (2)	O2—O4	3.061 (3)
F1—F2^i^	2.736 (3)	O2—O3^iii^	3.113 (4)
F1—O1	3.051 (4)	O3—O3^iii^	3.128 (4)
F2—O1	2.679 (3)	O4—O4^ix^	2.768 (4)
F2—F3^i^	2.714 (3)	O4—O5^x^	2.793 (3)
F2—F3	2.759 (3)	Cu—O3^iii^	2.715 (3)
F2—O4^iv^	2.766 (3)	Cu—Cu^iii^	3.575 (3)
			
F4—Cu—O1	86.42 (9)	F1^i^—Fe—F2^i^	89.43 (9)
F4—Cu—O2	89.89 (8)	F1—Fe—F3	90.08 (7)
O1—Cu—O2	171.49 (9)	F1^i^—Fe—F3	89.92 (7)
F4—Cu—O3	173.09 (9)	F2^i^—Fe—F3	89.06 (9)
O1—Cu—O3	91.47 (9)	F2—Fe—F3	90.94 (9)
O2—Cu—O3	91.30 (8)	H11—O1—H12	109 (3)
F4—Cu—O4	89.27 (9)	H21—O2—H22	104 (3)
O1—Cu—O4	97.50 (10)	H31—O3—H32	98 (3)
O2—Cu—O4	90.11 (9)	H41—O4—H42	103 (4)
O3—Cu—O4	97.53 (9)	H51—O5—H52	102 (3)
F1—Fe—F2^i^	90.57 (9)		

Symmetry codes: (i) −*x*, −*y*, −*z*; (ii) −*x*+1, −*y*, −*z*; (iii) −*x*+1, −*y*+1, −*z*; (iv) −*x*+1, −*y*+1, −*z*+1; (v) *x*, *y*−1, *z*; (vi) *x*−1, *y*, *z*; (vii) −*x*, −*y*+1, −*z*; (viii) −*x*+1, −*y*+2, −*z*+1; (ix) −*x*+2, −*y*+1, −*z*+1; (x) *x*+1, *y*, *z*.

### Hydrogen-bond geometry (Å, °)

**Table:**

*D*—H···*A*	*D*—H	H···*A*	*D*···*A*	*D*—H···*A*
O1—H11···F1^ii^	1.00 (4)	1.59 (4)	2.590 (3)	173 (4)
O1—H12···F2	0.99 (4)	1.71 (4)	2.679 (3)	167 (4)
O2—H21···F1^iii^	1.02 (5)	1.61 (5)	2.595 (3)	161 (4)
O2—H22···O5^viii^	0.98 (4)	1.82 (4)	2.699 (3)	147 (5)
O3—H31···F3^iii^	1.07 (5)	1.52 (5)	2.577 (2)	167 (5)
O3—H32···F3^x^	1.04 (5)	1.64 (5)	2.668 (3)	171 (5)
O4—H41···F2^iv^	1.02 (4)	2.07 (9)	2.766 (3)	123 (8)
O4—H42···O5^x^	1.01 (9)	1.81 (9)	2.793 (3)	164 (8)
O5—H51···F2^xi^	1.01 (6)	2.05 (7)	2.888 (3)	139 (5)
O5—H51···F3^vii^	1.01 (6)	2.23 (5)	3.072 (4)	140 (6)
O5—H52···F4	1.05 (7)	1.63 (7)	2.676 (3)	175 (6)
F4—H6···F4^iv^	0.91	1.82	2.597 (4)	142

Symmetry codes: (ii) −*x*+1, −*y*, −*z*; (iii) −*x*+1, −*y*+1, −*z*; (viii) −*x*+1, −*y*+2, −*z*+1; (x) *x*+1, *y*, *z*; (iv) −*x*+1, −*y*+1, −*z*+1; (xi) *x*, *y*+1, *z*; (vii) −*x*, −*y*+1, −*z*.

### Table 3 Valence-bond analysis according to the empirical expression from Brown &amp; Altermatt (1985), using parameters for solids from Brese &amp; O'Keeffe (1991)

**Table:**

	O1	O2	O3	O4	F4	F1	F2	F3	O5	Σ	Σ expected
Cu	0.49	0.47	0.45	0.16	0.44					2.01	2
Fe						0.51 (x2)	0.49 (x2)	0.48 (x2)		2.96	3
H11	0.80					0.20				1	1
H12	0.80						0.20			1	1
H21		0.80				0.20				1	1
H22		0.80							0.20	1	1
H31			0.80					0.20		1	1
H41				0.80			0.20			1	1
H42				0.80					0.20	1	1
H51							0.10	0.10	0.80	1	1
H52					0.20				0.80	1	1
H6					0.80 (x0.5)					1	1
					0.20 (x0.5)						
Σ	2.09	2.07	2.05	1.76	1.14	0.91	0.99	0.98	2.00		
Σexpected	2	2	2	2	1	1	1	1	2		
